# Chronic Disease in the Community (CDCom) Program: Hypertension and non-communicable disease care by village health workers in rural Uganda

**DOI:** 10.1371/journal.pone.0247464

**Published:** 2021-02-25

**Authors:** Joseph H. Stephens, Aravind Addepalli, Shombit Chaudhuri, Abel Niyonzima, Sam Musominali, Jean Claude Uwamungu, Gerald A. Paccione

**Affiliations:** 1 Kisoro District Hospital, Kisoro, Uganda; 2 Doctors for Global Health, Decatur, Georgia, United States of America; 3 Albert Einstein College of Medicine/Montefiore Medical Center, New York, New York, United States of America; University of the Witwatersrand, SOUTH AFRICA

## Abstract

**Background:**

Although hypertension, the largest modifiable risk factor in the global burden of disease, is prevalent in sub-Saharan Africa, rates of awareness and control are low. Since 2011 village health workers (VHWs) in Kisoro district, Uganda have been providing non-communicable disease (NCD) care as part of the Chronic Disease in the Community (CDCom) Program. The VHWs screen for hypertension and other NCDs as part of a door-to-door biannual health census, and, under the supervision of health professionals from the local district hospital, also serve as the primary providers at monthly village-based NCD clinics.

**Objective/Methods:**

We describe the operation of CDCom, a 10-year comprehensive program employing VHWs to screen and manage hypertension and other NCDs at a community level. Using program records we also report hypertension prevalence in the community, program costs, and results of a cost-saving strategy to address frequent medication stockouts.

**Results/Conclusions:**

Of 4283 people ages 30–69 screened for hypertension, 22% had a blood pressure (BP) **≥**140/90 and 5% had a BP **≥** 160/100. All 163 people with SBP **≥**170 during door-to-door screening were referred for evaluation in CDCom, of which 91 (59%) had repeated BP **≥**170 and were enrolled in treatment. Of 761 patients enrolled in CDCom, 413 patients are being treated for hypertension and 68% of these had their most recent blood pressure below the treatment target. We find: 1) The difference in hypertension prevalence between this rural, agricultural population and national rates mirrors a rural-urban divide in many countries in sub-Saharan Africa. 2) VHWs are able to not only screen patients for hypertension, but also to manage their disease in monthly village-based clinics. 3) Mid-level providers at a local district hospital NCD clinic and faculty from an academic center provide institutional support to VHWs, stream-line referrals for complicated patients and facilitate provider education at all levels of care. 4) Selective stepdown of medication doses for patients with controlled hypertension is a safe, cost-saving strategy that *partially* addresses frequent stockouts of government-supplied medications and patient inability to pay. 5) CDCom, free for village members, operates at a modest cost of 0.20 USD per villager per year. We expect that our data-informed analysis of the program will benefit other groups attempting to decentralize chronic disease care in rural communities of low-income regions worldwide.

## Introduction

### General

In 2017, non-communicable diseases (NCDs), primarily cardiovascular diseases (CVDs), cancer and chronic respiratory disease, were responsible for 74% of deaths worldwide, with over three quarters of NCD deaths occurring in low- and middle-income countries [1]. Hypertension, the largest modifiable risk factor in the global burden of disease, is prevalent in sub-Saharan Africa, although rates of awareness and control are very low, particularly in rural areas [2–6].

In the 2014 national NCD survey in Uganda, 70% of participants (ages 18–69) had never had their blood pressure (BP) measured. Of those with >30% 10-year risk of developing CVD, only 13% had been treated or counseled [7]. Along with low rates of awareness, other important barriers to NCD management in Uganda include workforce shortages, provider knowledge deficits, lack of access to affordable medications and underfunding [8–10]. Uganda has only 1.2 physicians and 13.1 nurses per 10,000 people, most of whom work in urban areas, and providers at all levels lack the training and confidence to adequately treat NCDs [10]. Although medications supplied by the National Medical Store (NMS) are available at government health facilities free-of-charge, stockouts of NCD drugs are common, particularly at lower-level health centers [9, 11]. In 2014, the Department of Community Health, which includes the Programme for the Prevention and Control of NCDs and 9 other divisions, received only 0.37% (902,000 USD) of the overall Ministry of Health (MOH) Budget [10]. As of the 2017–2018 financial year, this allocation was largely unchanged; however, in 2019, the NCD Programme was designated a Department of the MOH, and funding may increase [12, 13].

The dual constraints of provider shortages and underfunding have led to significant interest in task shifting strategies, which, for NCDs, requires revising traditional episodic delivery of care. In a 2019 systematic review and qualitative analysis, Heller et al. identify several factors that facilitate shifting NCD care to nonphysician health workers (NPHW) [14]. These factors include NPHW residence in the community served; detailed, ongoing training and supervision; autonomy in prescribing and decision-making; reliable systems to track patient data; uninterrupted supply of medications; and adequate compensation.

A growing body of literature suggests that task shifting CVD interventions to nurses and community health-workers (CHWs) in low- and middle-income countries can indeed accomplish important objectives. Specifically, CHWs have been able to screen for hypertension and elevated CVD risk in the community, link new hypertensives with care, educate and monitor patients, improve BP and reduce CVD risk in uncontrolled patients, modify lifestyle and behavior, and improve adherence [15–27]. Most studies were of relatively short duration (months to 2 years) and focused on one or two stages of the care continuum. No reports describe a CHW-orchestrated, multi-year program for NCDs—meeting Heller, et al.’s quality criteria cited above—that starts with comprehensive, home-based screening and leads to village level management of multiple chronic diseases. This study addresses that gap.

### Site and program background

Kisoro District in Southwestern Uganda has a population of 282,000. In the government census of 2014, 86% of people in Kisoro district were subsistence farmers, 69% of adults were married, 86% had not passed grade S4, and 40% were illiterate. Since the government census includes Kisoro town, it is highly likely that in the remote rural villages where our program operates, over 95% are subsistence farmers, and many more are illiterate, officially unmarried, and less educated than these district-wide census data suggest. Three quarters of the Kisoro population live within 5km of the nearest lower-level health center (HC II, III or IV). However, there is limited chronic disease care at these facilities, and the few NCD medications are often unavailable. Since 2007, local village health workers (VHWs) have been active in the district as part of a program sponsored by Kisoro District Hospital (KDH), a community-based NGO known as Doctors for Global Health (DGH), and the Albert Einstein College of Medicine/Montefiore Hospital in New York. The VHW program, supported by modest external funding, has a much broader portfolio of activities than Uganda’s public-sector volunteer Village Health Teams (VHT). The VHWs provide prevention, clinical care, and education in 6 domains of health (child wellness, women’s health, NCDs, sanitation and hygiene, acute illness and community health) and are supervised twice a month in their villages by clinical officers or registered nurses from KDH. The VHWs go door-to-door in their villages and screen for hypertension and other NCDs every 2 years. Since 2011, the VHWs also manage patients monthly in village-based clinics through a project called Chronic Disease in the Community (CDCom). Originally operating in a handful of villages in 1 sub-county, CDCom has since expanded to 52 villages in 2 sub-counties. The CDCom program is the subject of this analysis [14].

In the first report about the CDCom program, O’Neil et al. described VHW demographics and the details of the VHW program, including its selection process, training courses, certification and continuing education [22]. The paper evaluated access to care and the quality of hypertension control in CDCom in 2013 as compared to a control cohort of patients from the district hospital chronic disease clinic. The concluding results: CDCom patients were evaluated and received medications more regularly, every 35.6 days vs. every 50.8 days. CDCom patients had a mean systolic blood pressure (SBP) of 148 vs. 157 and were more frequently measured at or below SBP goal (71% vs. 60%).

## Objective

Whereas O’Neil, et al demonstrated the efficacy and feasibility of managing hypertension in the field with VHWs, the objective of this report is to describe—within a 15-year old VHW program defined by the parameters outlined by Heller—the decade-long *operation* of the CDCom program, from the comprehensive population-based identification of hypertensive patients to the logistics of village-based NCD care. We describe patient enrollment and VHW incentives to screen and identify patients; the growth, present size and composition of CDCom; risk-based approach to hypertension treatment; logistics of where, when and how care is delivered; the natural link between community and district hospital care through supervision; methods of documentation and consultation; treatment strategies during stockouts of government supplied drugs and estimates of program cost.

We expect that our data-informed analysis of the program will benefit other groups attempting to decentralize chronic disease care in rural communities of low-income regions worldwide.

## CDCom program description

### CDCom foundations

The DGH-Einstein-KDH collaboration sponsors 3 allied projects that have provided a critical foundation for the CDCom program:

- Chronic Care Clinic (CCC): In 2006, the collaboration established the CCC at KDH. The CCC is the facility-based “parent” of CDCom and the first and only publicly-financed clinic in the district delivering continuous care to patients with hypertension, diabetes, heart disease, asthma, epilepsy and other NCDs [28]. Patients are referred to the CCC from the KDH wards after hospital discharge or from private and lower-level public clinics, which do not consistently treat NCDs. Patients travel an average of 8km to the CCC, usually on foot. Alternatively, a roundtrip on a motorbike-taxi (boda), costs a day’s pay. As of early 2020, with a team of 5 Ugandan providers, clinical officers and nurses, and rotating senior medicine residents from Montefiore, the CCC cares for over 1400 active patients—800 with hypertension. All providers are precepted by Montefiore “Global Health and Clinical Skills” (GHACS) faculty each of whom attends 2–3 months annually at KDH and helps cover its internal medicine service throughout the year. Despite its growth and position as the dominant provider of chronic disease care in the district, the CCC reaches only 20–40% of *untreated* hypertensives with SBP >170. The CCC welcomes all, although distance and poverty severely limit access.
- Biannual Health Census and Population-based Blood Pressure screening: Since 2012, the VHW program has sponsored a comprehensive biannual health census of *every* home by a trained project assistant and each village’s VHW. Performance of the health census is supported by KDH leadership and the village chairperson and complements without duplicating the data available in the government census.
  The program’s *health* census gathers data on various dimensions of health used by the VHW in serving the family and larger community: family demographics and recent changes, child health indices, women’s health (e.g. cervical cancer screening, family planning), chronic disease, high-risk families (e.g. 3 children under 5 years, malnutrition, solitary elder, child-headed, poor sanitation), environmental health and interest in various health topics. The health census takers survey 14–16 homes per day after receiving verbal consent (nearing 100%) from a competent adult in each home (usually the maternal or paternal head-of-household).
  Of relevance to the CDCom program, during the census the VHW screens for hypertension by measuring the BP of every adult older than 25 years. If the SBP is >140, the individual is designated for biannual follow-up assessments. If the SBP >160, it is taken again by the project assistant and the following month by a clinical supervisor from KDH. If the BP meets enrollment criteria of >169 (see below, CDCom Hypertension Treatment Strategy), the patient is referred to CDCom, where their BP is taken a third time prior to enrollment. For adults that are not home during census time, the VHW is expected to return once for a BP check if the individual’s BP was elevated on a previous census or was not taken. Thus, broad, recurrent, population-based screening for this asymptomatic disorder is central to the CDCom program.
  The VHWs have been with the program for a mean of 8 (range, 3–13) years. Every VHW has been trained and “certified” to take BP with a calibrated aneroid manometer and stethoscope prior to participating in hypertension evaluations. Checking BPs is one of the most frequent diagnostic interventions the VHWs perform. VHWs are instructed to have the patient seated for minutes prior to taking the BP with the arm at rest and stethoscope at heart level. If SBP >140, it is repeated minutes later, and both readings are recorded. For clinical decisions in CDCom, the average is considered the BP of the visit; in Census, the second reading (or project assistant’s re-check) is recorded.
- VHW Performance-based Incentive System: Since 2010, the program has employed performance-based incentives: VHWs do not receive a base pay and are instead compensated for the performance of various activities which are validated by their supervisors twice a month [29]. Stipend amounts, varied seasonally to incentivize performance during certain periods (e.g. identifying malnourished children in peak months prior to harvests), are based on an estimate of each activity’s incidence and public health or clinical importance, and the effort required of the VHWs. New case identification of patients with chronic diseases such as hypertension receive above-average remuneration (2 USD/patient), and the VHW is thereafter given a monthly stipend of 0.35 USD for every patient attended to in CDCom.

### CDCom patient enrollment

**Fig 1:**
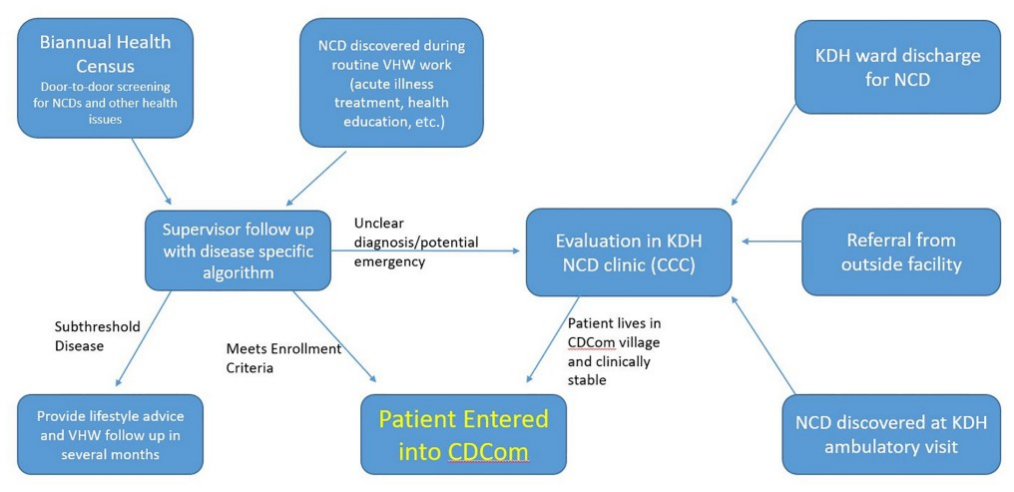
Referral pathways to CDCom. Routes of patient entry into CDCom. CCC: Chronic Care Clinic; CDCom: Chronic Disease in the Community program; KDH: Kisoro District Hospital; NCD: non-communicable disease; VHW: village health worker.

Patients are referred to community CDCom sites from several sources—most importantly, the door-to-door biannual health census mentioned above, but also from KDH’s inpatient wards, the CCC and other health facilities (Fig 1).

Between 2011 and 2015, most patients were referred to CDCom from the CCC. Many of these patients were initially diagnosed with hypertension during a hospitalization or ambulatory visit at KDH and cared for in the CCC prior to the creation of CDCom. To transfer to CDCom, patients had to have SBPs <170 for 6 months, good adherence to medications and no recent cardiovascular complications. Patients recently hospitalized and followed in the CCC until stable can be transferred as well.

Since 2016, VHWs identify most new CDCom enrollees during the census (in either its first or second iteration in all villages) or other home visits, and, as described above, are incentivized to identify hypertensives and others with NCDs.

When validating hypertension (and other) diagnoses in the field, the supervisor follows disease-specific scripted enrollment forms to focus history-taking and perform examinations (e.g. BP, urinalysis, blood glucose). Each form contains a simple algorithm which guides the supervisor to either directly enroll the patient into CDCom, refer the patient to the hospital for evaluation of unclear diagnoses or potential emergencies/complications, or provide lifestyle advice and have the CHW follow up mild disease after a period of months (S1 Appendix).

The third source of enrollment in CDCom is through referral from clinics other than KDH after the patient relocates residence to a CDCom village. In this case, the patient must be evaluated in the CCC to verify records and criteria for CDCom eligibility. They are then paired with the proper CCC provider (the clinical supervisor of the patient’s village—see below), and a CCC medical record is established.

### CDCom population

With increasing community awareness and coverage, CDCom has grown substantially. However, despite a catchment area of ~49,000 people, CDCom still covers only ~15% of the villages in the Kisoro District. The 200 patients enrolled in 2015 increased to 538 during 2017 with the addition of 10 new villages to the existing 42. By December 2019, this number rose to 761, with a monthly average of 9 new enrollees, 3 people dropping out or moving, and 1 death.

Currently, 721 patients carry a single diagnosis: 373 have hypertension, 184 asthma, 100 epilepsy, 32 diabetes, 22 mental health disorders, 5 (non-hypertensive) congestive heart failure, 1 Parkinson’s disease, 1 “arthritis,” 2 sickle cell disease, and 1 hyperthyroidism. Forty patients carry more than 1 diagnosis, all of whom have hypertension: 17 with comorbid diabetes, 12 heart failure, 10 asthma, and 1 with hypertension, epilepsy and diabetes. Seventy five percent of all patients and 75% of hypertensives are female.

Patients live an average of 8km from the CCC and, before CDCom, would have to travel 16km roundtrip by foot or motorcycle taxi to access routine chronic disease care. Instead, they walk a mean of 0.67km from home to a CDCom meeting point (1.3km roundtrip), or their VHW sees them at home.

### CDCom hypertension treatment strategy

Although Ugandan national guidelines for hypertension recommend prompt treatment of BP>160/100 and of BP >140/90 after a 2-month trial of lifestyle modification, the poverty and frequent stockouts of government drugs makes the 140/90 threshold unfeasible and potentially unhealthful for our population. To adapt to scarce resources, we have adopted a risk-based therapeutic strategy that maximizes cost-effectiveness by prioritizing CVD risk over absolute blood pressure thresholds. Both approaches have advantages: numerical thresholds are easy to use, while, given the generally lower prevalence of atherogenic risk factors in this agrarian African community, a risk-based approach implies initiating treatment at higher levels of BP, treating fewer patients, requiring fewer providers, and allocating drugs to those who would benefit most.

The following is excerpted from a recent report on the CCC [28]:

… The CCC [and CDCom] has used the WHO CV risk prediction charts (2007) for “AFR E” region and treats those with a 20% or greater 10-year risk of CVD. For example, patients 60 years and older with an SBP >180 have at least a 20% risk, while those 40–60 years have a 10–20% risk. In practice, … (we chose) a lower threshold of treatment for all patients with uncomplicated hypertension—SBP >170 (measured at least twice on 2 separate clinic visits) … a risk below 20%. The other exception to the “above 20%” rule is that all patients with stage 1 hypertension and either diabetes, kidney disease or complications of CVD are treated even if the 10-year risk is <20% [threshold, SBP>140]. CCC does not treat non-diabetic patients with SBP less than 170 regardless of age or smoking status, as none of these patients have 10-year CVD risk greater than 20%. Once treatment is initiated for uncomplicated hypertension, the target SBP is ≤159, chosen as 10 points below the threshold of treatment.

If the SBP is 140–169, the patient is given lifestyle advice and followed up regularly by the VHW for a year. If the threshold of SBP >169 is reached, the patient is enrolled in CDCom.

Besides being pragmatic, the higher-threshold strategy may be more salubrious for a poor population with few atherogenic risk factors and very limited access to care or laboratory monitoring: treatment side effects are minimized, while social resources are preserved for competing goals such as nutrition and education [30].

### CDCom care team: Unique continuity

At CDCom, VHWs partner with a KDH-based supervisor, one of the 4 mid-level providers in the CCC whose job definitions are purposefully constructed to facilitate continuity of care between the 2 chronic disease programs. Supervisors do not rotate CDCom posts and over time become familiar with their entire CDCom patient panel. When clinical complications or questions arise that require consultation, the supervisors *refer the patient to themselves* in the CCC and seek the input of the GHACS faculty preceptor. Consultation is efficient and personal, supervisor learning is emphasized, and the patient benefits from continuity across sites.

During the first years of CDCom, VHWs were primarily responsible for mobilizing patients to the community clinics, which were run by supervisors. Over the past 2 years, the program has shifted and the clinic is now run by VHWs and precepted by supervisors (see below).

### CDCom logistics of place, time, clinical encounter, and consultation backup

Patients and VHWs from 1 to 5 villages convene at central meeting points once per month. The supervisor, carrying lab equipment (glucometer, urine analysis kit, electronic scale) and patient-specific packets of medications, visits 3 sites per day spending 1–3 hours at each, depending on the number of patients enrolled at the site. Thus, all 52 villages are visited over the course of 12 supervisor workdays, or “CDCom sets.”

Prior to the supervisor’s arrival, VHWs follow scripted guidelines to interview patients regarding medication adherence and symptoms. They also measure SBP for those with hypertension, diabetes or heart failure (S2 Appendix). Then, with the patient present, the VHW presents the relevant clinical data to the supervisor, who corroborates abnormal measures and takes additional history as needed. The VHWs *and* supervisors *both* record the clinical data in identical patient-specific forms. The forms are sectioned by disease and highlight relevant symptoms, exam signs and drug adherence (S3 Appendix). The VHW keeps one copy for potential follow-up between CDCom clinics, and the supervisor returns to the hospital with the second copy.

**Fig 2:**
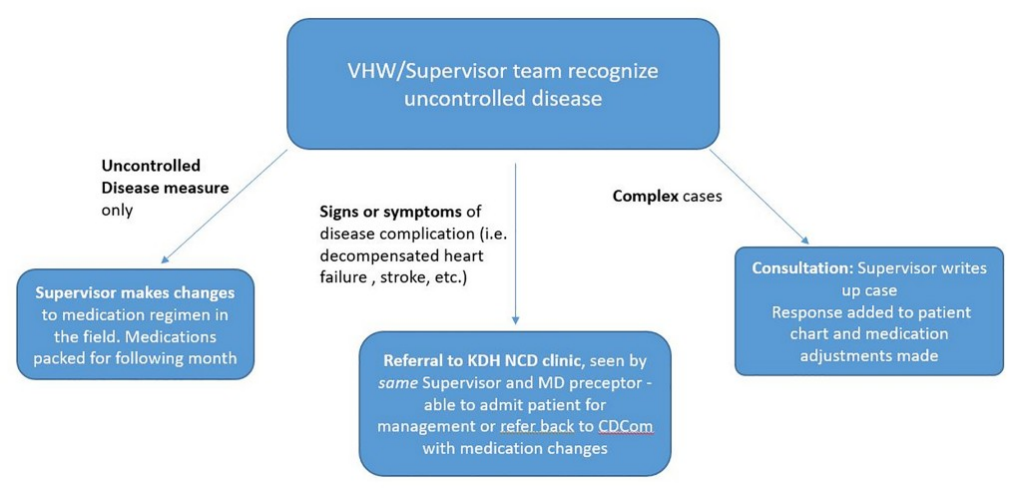
Management of unstable disease. Supervisor options for management of unstable disease measures or symptoms and signs of complications. CDCom: Chronic Disease in the Community; KDH: Kisoro District Hospital; NCD: non-communicable disease; VHW: Village Health Worker.

Unstable CDCom patients are managed in one of three ways (Fig 2):

- For patients who are asymptomatic but with uncontrolled SBP or serum glucose, medication *adjustments are determined by the supervisor* in the field, indicated in the chart, and implemented the following month when medications are repacked. The VHW is incentivized with a modest stipend to follow up at the patient’s home in the interim to collect additional measures, assess adherence, and (very rarely) refer the patient to the hospital.
- With more complicated but asymptomatic patients whose measures are *persistently* out of control or otherwise clinically challenging, *charts are flagged by the supervisor*. The flagged charts are reviewed monthly by a designated CCC-based clinical officer who composes synopses of the patients’ histories, treatments and measures and sends them to program faculty in NY. The returned consultations are then inserted into the patient chart for review by the supervisor and VHW. Five or six of these write-ups also become the grist for twice monthly continuing education sessions for *all* supervisors, thus enhancing NCD knowledge through case-based teaching.
- Patients with concerning new cardiovascular **(**or other**)** *symptoms* are *referred by the supervisor promptly to him/herself* in the hospital-based CCC for in-person consultation with an attending GHACS physician, as mentioned above.

### Medications and the problem with stockouts

A large portion of the CDCom budget is earmarked for medications. Ideally, drugs are allocated to CDCom by KDH which incorporates chronic disease medications for CCC *and* CDCom into the hospital requisitions submitted quarterly to the National Medical Stores (NMS). However, budget restrictions limit drug orders. Often, not enough are ordered to meet hospital needs, less than 70% of orders are filled, and therapies vie with each other (e.g., antibiotics versus anti-hypertensives). Thus, stockouts of important medications, including first line antihypertensives, are frequent, and the program must buy drugs from private retailers. While NMS supports most chronic disease medication (50–80%) used by CDCom patients, DGH has supported ~80% of the *hypertensive* medication prescribed in the program.

Over the past 5 years, unrestricted funding for a growing CDCom became untenable for DGH. When discussed with patients, few were willing to pay a portion of the medication cost. As one elderly woman said, “First you tell me I have a sickness I don’t feel, and then you want to charge me for treating it?” Therefore, to provide reliable access to medication while reining in program costs, a *drug prioritization strategy* based on patient *risk* was instituted during stockout periods. Patients were placed into one of three groups according to disease severity/comorbidity, and medications stepped down in the 2 less severe subgroups (see S4 Appendix). The patients with severe hypertension (SBP >175**)** or those with cardiovascular complications were given full doses of all prescribed meds. Patients could buy the remainder of their originally prescribed dose as desired, although very few did. The cost savings and control achieved with this strategy are presented in Results.

### Data analysis

Data for community hypertension prevalence comes from the program’s first-time biannual health censuses (2012–2016, described above), performed prior to or concurrent with community enrollment in CDCom. To facilitate comparison with the standardized WHO “STEPwise” approach (STEPS), Kisoro rates are age-adjusted as per STEPS. Hypertension was defined as measured elevated blood pressure at thresholds of 140/90 and 160/100, or a self-report of a prior diagnosis of hypertension. To account for the possibility of blood-pressure lowering treatment, those with a prior diagnosis were included in the **≥**160/100 group regardless of measured blood pressure as it was not assessed if they were taking medication at the time of screening. Individuals meeting criteria for the **≥**160/100 group were also counted in the **≥**140/90 group.

The bulk of this study is a descriptive analysis of CDCom, with a focus on hypertension—the most common disorder in the program (~50% of patients). We report the linkage rate (the proportion of patients found to be hypertensive in the census that received follow up) and the retention rate for two example years (the proportion of patients who begin and end a 12-month period enrolled in CDCom). The analysis is supported by a retrospective review of the CDCom electronic patient registry from January 2016 through February 2017, and, for a more recent perspective, the calendar year 2019. The 2016–17 data allow for some comparisons (see “stockout strategy” below) not available subsequently. We also present data comparing rates of hypertension control and medication costs for patients before CDCom’s “stockout strategy” was initiated (January–May 2016) and after it was universally practiced in all villages (December 2016–February 2017). We then report the extent and costs of the stockout strategy for the year 2019. Medication costs are calculated based on wholesale prices from local pharmacies and include all medications whether supplied by the National Medical Stores (NMS) or bought wholesale by the program. All data is publicly available on an online repository [31–33].

### Ethics statement and funding note

The Albert Einstein College of Medicine IRB and the Kisoro District Hospital Administrative Committee approved this study (# 2017–8404). Consent was not obtained as data was analyzed retrospectively and anonymously. Of note, all data are secondary, part of the program’s ongoing quality assessment and based on monthly and annual reports of program data.

## Results

### A. Local vs national hypertension prevalence

Between 2012 and 2016, nearly 4,300 residents in 43 rural villages of Kisoro district (estimated to be >85% of the adults 30–69 years) were screened for hypertension. Thirty two percent were male. The prevalence of at least stage 1 hypertension (BP**≥** 140/90) was 22% and stage 2–3 hypertension (BP**≥** 160/100), 5%. While males were more likely than females to have stage 1 hypertension (26% vs 20%), the rate of stage 2 hypertension was 5% in both sexes. One percent of those screened had been previously diagnosed with hypertension and 84% with SBP >160 were unaware of having hypertension.

### B. CDCom logistics: Linkage with census, patient demographics, enrollment source, attendance, degree of BP control and retention

Of 163 people who had an SBP >169 during the census, 91 were eventually enrolled in CDCom, while 23 were documented with an SBP <170 on repeat exam. Nine patients had no follow up. VHW’s reported that an additional 40 people were followed and did not meet criteria for CDCom enrollment, but there was no documentation. Thus, depending on the reliability of the VHWs memory of following up and finding the lower BPs they recall, 70–95% of persons with SBP >170 were linked with some form of follow up.

At the end of 2019, 413 hypertensive patients were active in CDCom, mean age 61 (range, 28–91). Sixty-five (16%) are between 30 and 49 years, 237 (59%) between 50 and 69 years, and 101 (25%) are >69 years. Of hypertensive patients, 0.4% have diabetes, 2.7% have heart failure and 2.2% have asthma (the latter two clinically diagnosed).

One hundred eighteen hypertensive patients (29%) transferred into CDCom from the CCC when CDCom began in their village, 245 (59%) were first identified by VHWs during census home visits and enrolled in the field, and 50 (12%) were followed in another health facility before moving to a CDCom village or otherwise transferring care.

The mean group SBPs at enrollment (average of 2 same-day measures at the first meeting) varied by source, largely because the CCC and “other” transfers were being treated. The initial enrollment BPs of these groups are provided in Table 1.

**Table 1:** Mean CDCom enrollment BPs by enrollment source.

Enrollment Source	Mean Enrollment BP
CCC transfers (N = 118, 29%))	163/74mmhg
Field (N = 245, 59%)	181/94
Other (N = 50, 12%)	154/80
TOTAL (N = 413)	173/87

CCC, Chronic Care Clinic; Field, enrolled by VHW through Census or other village work; BP, blood pressure.

Table 2 shows the BP distribution, by age, for 403 of the 413 hypertensive patients enrolled in CDCom according to their last BP measure in 2019 (10 charts were unavailable). Three hundred and ninety of these were taken in December 2019, and 13 in November. Two hundred and seventy-three (68%) were at goal with an SBP ≤159 and 88 (22%) had a SBP ≤139. Thirty two percent of patients were not at goal, with an SBP of **≥**160mmHg and 14% **≥**170. Of the 7 hypertensives who died in 2019, all were older than 65 years, 6 were either octogenarians or had significant co-morbid disease (HIV, cancer, CHF), and all had BP at goal (mean SBP 143).

**Table 2:** Distribution of last BP measure of 2019 for 403 CDCom patients with hypertension.

SBP Range	ALL ages N (%)	Age 30–49 N (%)	Age 50–69 N (%)	Age >69 N (%)
**≥ 180 mmhg**	38 (9.4)	5 (7.7)	23 (9.7)	10 (9.9)
**170–179**	20 (5.0)	6 (9.2)	10 (4.2)	4 (4.0)
**160–169**	72 (17.9)	11 (16.9)	43 (18.1)	18 (17.8)
**159–140**	185 (45.9)	23 (35.4)	115 (48.5)	47 (46.5)
**≤139**	88 (21.8)	20 (30.8)	46 (19.4)	22 (21.8)

SBP, systolic blood pressure.

Monthly BP measures were documented 92% of the time, either at the CDCom site meetings, or by the VHW visiting the home. Most of these latter patients are elderly and cannot easily walk to the meeting point. Eight percent of the time, the patient was not seen, and the drugs were left with a family member.

The year-to-year retention rate of patients in CDCom is high: of the 273 patients enrolled in January of 2017, 236 (86%) were still active in CDCom in January 2018. Of 340 patients in January 2018, 337 (99%) were active in January 2019. The increase in patients between 2017 and 2018 is due to the addition of 11 new villages to CDCom in the Spring of 2017.

### C. Drug prioritization (Stockout) strategy, cost saving and blood pressure control

Three analyses are relevant to the strategy CDCom employs when the supply of medications is low: 1) comparison of BP control achieved before and after the strategy was initiated in 2016, 2) its extent and impact on BP control for the year 2019 and 3) cost implications.

#### 1) 2016 “Pre-Post” comparison

As of January 2016, 132 of the 162 hypertensives in CDCom were eligible for the “pre-post” comparison. Of the 30 ineligible patients, 9 had not come to CDCom meetings, 10 had moved, 4 died before January 2016, 2 were being trialed off medications, and 1 had been referred to CCC.

Fifty five percent (72/132) of hypertensive patients were categorized into the low and intermediate-severity groups. Of these patients, in the 5 months prior to the dose adjustment, the average patient BP was 146/83, while 21% of patients had an average SBP >160 mmHg and 10% had an average SBP >170 mmHg. In the 3 months after dose adjustments, the average patient’s BP in the low/intermediate group was 148/85 with 18% of patient’s SBP’s above 160 and 8% above 170 mmHg.

In the high-severity group (given full doses of all meds) during the “pre-period” from January to May 2016, the average patient BP was 152/86. Thirty two percent of patients had an average SBP greater than 160 and 20% an average SBP greater than 170. In the “post-period” from December 2016 to February 2017, the average patient BP was 156/87; 35% had an average SBP greater than 160 and 23% an average SBP greater than 170.

#### 2) 2019 drug prioritization (Stockout) strategy and BP control

By the end of December 2019, of the 403 hypertensive patients with accessible records, 160 (40%) were being treated with full doses of medication while 243 (60%) were maintained on lowered doses, following prioritization criteria, due to persistent shortages of anti-hypertensives.

The mean BP of the total 243 patients initially on stockout doses at the start of 2019 was 143/81. Sixteen (6.6%) had a BP rise above SBP 170 and resumed full-dose treatment at some point in the year. Their average BP had risen from 144/83 to 172/87. The other 227 (93%) on stockout doses for the year maintained SBPs between 100 and 160 and a mean for the last 3 BP measures in 2019 of 142/80.

For the 160 patients on *full-dose* treatment, the mean of the last 3 BP measures in 2019 was 159/86. Of these, 45 (28%) patients had a mean SBP >170 (i.e. BP 184/93), most of which were flagged, written up, and reviewed with faculty in NY. The mean BP of the other 115 patients was 153/84.

#### 3) Cost savings with the stockout strategy

2016: Pre-Post Comparison: In the month prior to the implementation of the dosage adjustment, the average wholesale price of medications for all hypertensive patients was 1.38 USD per patient per month. The price of hypertensive medications for those patients that met the stockout criteria was 1.04 USD per patient per month. For those that did not meet criteria, the cost was 1.75 USD per patient per month. After the dose adjustment period, the price for dose-adjusted patients dropped 57% to 0.45 USD per patient per month while the price for non-dose adjusted patients dropped to a lesser degree (32%, to 1.2 USD per patient per month).

2019: The average wholesale per-person cost of anti-hypertensives was 1.08 USD per month—0.86 USD for patients on stockout doses and 1.41 USD for full-dose patients. With dose adjustments, the cost reduced 0.52 USD per patient per month from what full dose would have been, a savings of 46% per individual stepped-down and 32% overall savings for the program.

### D. Cost efficacy of the CDCom program

In 2019, the program cost $9,990 USD (3,650 USh/1USD) and served a catchment area of ~49,400. With the comprehensive screening of the population through the biannual census and VHWs’ incentives to screen during the year, the program likely covers the vast majority of hypertensives with SBP >170 in our 52 villages. CDCom costs 0.20 USD (730 USh) per year per inhabitant covered by the program.

Sixty percent of the budget is allocated to drug purchases, 14% to VHW stipends, 12% to supervisor stipends, 10% to administration/education (planning, record keeping, medication requisition/packaging, data analysis, consult coordination/education), and 5% motorcycle fuel and maintenance.

## Discussion and conclusions

In this report, we have presented a comprehensive model for VHW-led NCD screening and management, including novel strategies that address common barriers to care such as lack of awareness, access, provider training, consistent record keeping and medication stockouts. We make five principle contributions to the literature.

### 1) Lower rates of hypertension in rural Kisoro, a large but shrinking epidemiological divide

Our village-level screening results from 2012–2016 show a significantly lower hypertension prevalence in rural Kisoro than in the nation-wide STEPS survey, as demonstrated in Table 3. In 2014, Uganda performed a national NCD survey that employed the STEPS approach [7, 34]. In STEPS, hypertension was defined by either measured elevated blood pressure (≥140/90 stage 1, ≥160/100 stage 2) or taking prescribed medications for hypertension within the past 2 weeks. People taking BP medication within the past 2 weeks were classified as stage 2. The STEPS study screened 2,324 adults ages 30–69 (40% male), 27% of whom lived in urban areas, and found prevalences of stage 1 and 2 hypertension of 33% and 13% respectively. Thus, the prevalence of stage 1 hypertension was 33% lower in Kisoro than in STEPS, and stage 2 hypertension, 62% lower. Rates of prior diagnosis of hypertension in STEPS (11% of adults) were low, but significantly higher than in Kisoro (1%). Only 26% of those previously diagnosed with hypertension in STEPS were actively taking medications. Thus, in Kisoro, our assignment of 100% of those with a prior diagnosis of hypertension to the ≥160/100 category without regard to medication use likely overestimated the local prevalence of hypertension by ~0.75% (absolute proportions). Despite this overestimation, the absolute difference in prevalence (5% in Kisoro vs 13% nationally for SBP ≥ 160 and/or DBP ≥ 100 or on medications) is substantial.

**Table 3:** Hypertension prevalence in Uganda and Kisoro DGH VHW program villages.

	Uganda 2014 Nationwide STEPS Survey (mean BP 128/83)			DGH/KDH Census 2012–16 of 43 Villages (mean BP 120/75)		
	Total Surveyed	SBP ≥ 140 and/or DBP ≥ 90 or on meds	SBP ≥ 160 and/or DBP ≥ 100 or on meds	Total Surveyed	SBP ≥ 140 and/or DBP ≥ 90 or prior dx of HTN	SBP ≥ 160 and/or DBP ≥ 100 or prior dx of HTN
**Age Group**	N	**%**, N	**%**, N	N	**%**, N (% Change vs STEPS)*	**%**, N (% Change vs STEPS)*
**30–49**	1646	**26%**, 421	**8%**, 135	2788	**17%**, 465 (35% lower)*	**3%**, 72 (63% lower)*
**50–69**	678	**47%**, 317	**23%**, 158	1495	**32%**, 475 (32% lower)*	**10%**, 144 (57% lower)*
**30–69**	2255	**33%**, 737	**13%**, 293	4283	**22%**, 931 (33% lower)*	**5%**, 207 (62% lower)*

SBP, systolic blood pressure; DBP, diastolic blood pressure; meds, medications; dx, diagnosis; HTN, hypertension.

* Numbers in parentheses show the percent difference in proportion of individuals with elevated blood pressure (or on medications in STEPS, or with a previous diagnosis of hypertension in Kisoro) between Kisoro and Nationwide STEPS surveys. Note, HTN prevalence in Kisoro is likely overestimated (see Discussion).

Our findings are consistent with data from other rural areas of Africa, which have been spared the urban pressures of globalization and their associated atherogenic risks. Rural areas have demonstrated a relatively *low and static* prevalence of hypertension over decades [35]. Whereas CV epidemiology in urban areas is transitioning rapidly toward ischemic disease, the literature (and our Kisoro experience) suggests that in rural areas, the CV burden from atherosclerosis is still considerably lower than in the cities. Yet, CV morbidity and mortality from stroke or hypertensive cardiomyopathy is quite high in rural areas, largely due to difficult access to resourced health facilities that can detect or competently manage pre-morbid severe hypertension [35]. Although hypertension may be less prevalent, most hypertensive patients in rural Africa live their lifetime without being diagnosed or treated before presenting with an end-organ complication.

Furthermore, as suggested by more recent program censuses in our catchment area, the prevalence of hypertension may be increasing in parallel with development, motorized transport, and a Western diet. Combined with rapid population growth, a surge in atherosclerotic disease in the coming decades seems inevitable.

### 2) VHW’s providing a continuum of care from screening to management and/or referral

We show that VHWs, with proper training, supervision, and remuneration for their performance, can serve as frontline providers for patients with NCDs. As village members, VHWs are uniquely situated to screen systematically for “silent” NCDs before lethal complications supervene, and be the primary providers in rural areas of the developing world, thus mitigating the impact of the “workforce crisis.” As peers, they connect closely to patients and families whose understanding of chronic disease is limited, delivering medications to homes when necessary. Moreover, VHWs achieve high rates of linkage and retention in care and reasonable rates of BP control, affordably.

### 3) Unique linkages are feasible between data and care, hospital and community, and patients and NCD education of providers—Enhancing both population health and personalized care

Traditionally, public projects are divorced from clinics, and hospitals are disconnected from rural outposts. Population data are collected regularly by departments of public health and transmitted upstream to central governments to inform regional and national reports—but not the care of individual families or patients with specific diseases.

Taking a different approach, the CDCom model emphasizes *continuity* between domains of health that usually operate in isolation:

Link #1. Population-Clinic: VHWs go door-to-door collecting population health data and screening for disease to inform clinical practice: counseling about prevention and facilitating personal health interventions in malnutrition, geriatrics, ante-natal care, and CDCom—programs that they themselves, as VHWs, orchestrate. With clinical initiatives rooted in community-based health data, far-broader identification and earlier treatment of disease is possible than can occur within health facility walls.
Link #2. Community-Hospital: The VHWs, in turn, are supervised at CDCom by nurses and clinical officers who are also the providers at the district hospital’s chronic disease clinic, the CCC. The supervisors learn, first-hand, about NCD care in the community, and the VHWs learn from their supervisors’ NCD experience.
Link #3. Community-Consultation: When complications arise or consultation is needed, supervisors refer their CDCom patients *to themselves* in the CCC, where personal, on-site consultation is sought from board-certified internists.
Link #4: Consultation-Education: Continuing education of NCD providers, which usually occurs in classrooms, is “at the bedside” in the CCC and extends to CDCom. Learning is furthered by the composition of written case summaries by supervisors, which are emailed to experts in New York for written commentary and then become the grist of twice monthly educational sessions for the CCC-CDCom staff.

### 4) “Stepdown strategies,” which incur minimal increase in risk, save money and prioritize medications for the sickest patients

Since initiation of our drug prioritization (stockout) strategy, we have saved an average 40–60% of the cost of medications for patients whose doses were “stepped down.” This step-down regimen induced a minimal increase in mean SBP from 146 to 148 in 2016. In 2019, the mean SBP of 227 patients on step-down doses was 142. These findings parallel other studies that have successfully used step-down strategies for well-controlled hypertension [36, 37]. Furthermore, Law, et al., show that utilization of half of the recommended starting dose of first-line antihypertensives provides 80% of the BP lower effect of starting doses while substantially decreasing medication-related side effects [38]. We do not know the proportion of patients who supplement their lower doses by buying privately; however, since chronic disease medications are not commonly sold and our patients are very poor, private purchase is likely to be rare. In 2016, there also appeared to be collateral savings in medication costs in the full-dose group, likely due to more careful dose titration and attention to adherence by both patients and CDCom supervisors.

While the specifics of our step-down strategy may not be directly replicable in other settings, its success speaks to the relevance of flexible but monitored treatment guidelines that prioritize a limited supply of subsidized medication for those at highest risk of complications.

### 5) NCD care provided at a low overall cost

At a per-villager cost of 0.20 USD/year, the operating budget for our CDCom program is modest. When delivering NCD care as part of a comprehensive preventative and primary care service, like the VHW program, potential cost drops. Even after incorporating the cost of initial training, our overall VHW program—the care of acute illnesses, prenatal and newborn counseling and referral, targeted health education talks, door-to-door health census, CDCom providers (not drugs), and more—costs 0.72 USD/villager [29]. While these costs seem relatively low, scaling this kind of (intensive) program nationally for 42 million people would require a substantial increase in consistent government or third-party investment. Moreover, a major caveat in the cost of the CDCom program is our use of an SBP cut-off of 170 for uncomplicated hypertension, intentionally set to provide treatment only for those that reap the most benefit [28]. Treating lower risk patients would substantially increase overall costs.

### Limitations and scalability

One of the major strengths of CDCom and its data is also one of its most significant limitations: the program is fundamentally a *clinical* program, not a research project. The program has been embedded in the community for 10 years and successfully delivers care to a broad swath of the population. In doing so, the program depends on 52 VHWs and multiple project assistants to gather data accurately to inform ongoing evaluation and improve implementation. Heterogeneity of personnel introduces gaps and inconsistencies in the data over the years, from the methodology of measuring blood pressure to the completeness of the medical record, assessment of patient adherence, and compliance with program guidelines. Although all VHWs were instructed and “certified” in proper BP assessments and periodically re-evaluated, variability is introduced by a lack of digital devices employed by most hypertension prevalence studies (despite evidence that digital devices are also variable and overestimate BP), different models and years of the BP equipment, and varying experience among VHWs. A variety of errors that influence BP measures are undoubtedly made by our VHWs without being routinely corrected. Some tend to lower BPs erroneously: BP manometers go years without being calibrated, cuffs may be deflated too quickly, thin arms may be “over-cuffed,” and measures in treated patients are rounded down when close to 0. Other common errors tend to raise BPs: VHWs sometime perform screening assessments before the subject has been seated for 5 minutes; screened subjects sit on benches without back support; arms are often self-supported with stethoscopes below heart level; most BPs are taken only once, particularly if at goal; and subjects may be talking or listening while BP is taken. Moreover, financial incentives to identify new hypertensives may also lead to a tendency to overestimate BP. However, for a disease whose outcomes accumulate slowly over decades, and within a program that incorporates multiple BP assessments before either a diagnosis, payment, or a treatment change is made, most of these errors would be clinically insignificant.

To scale CDCom to a regional or national level, many potential pitfalls need to be avoided. First, as mentioned, CDCom flourishes within a larger “horizontal model” of village-wide delivery of multiple health interventions. Initiatives like CDCom are likely to be more costly when administered separately than within a comprehensive health package.

Second, leadership investment, often unmeasurable, is a critical component of any project. In Kisoro, the unique tripartite collaboration of Kisoro Hospital, DGH and Einstein/Montefiore has been highly invested for 14 years in the success of the VHW program and CDCom. Scale-up of such models is likely to succeed in places where the leadership is similarly invested, and the commitment is long-term.

Third, job definitions akin to those of our CDCom supervisors—nurses and clinical officers who are also the permanent primary providers in the hospital-based CCC—do not exist within the government system. Role consistency for nurses or clinical officers is rare in public facilities in which rotations among departments (and work shifts) regularly occur. The efficiency and cohesive personal care of CDCom are made possible by external funding for supervisor positions, with pay equal to government stipends.

Fourth, these clinical supervisors may be the most difficult group to replicate or replace, independent of funding source. We are presently training the top 20% of experienced VHWs as co-supervisors to oversee and teach junior cohorts of VHWs. Partnerships with veteran RN-CO supervisors—who alternate VHW supervision with the co-supervisor—could still maintain the CDCom-CCC linkage discussed above while facilitating at least local expansion of CDCom to surrounding villages.

Finally, cost-sharing with patients, which might substantially lower the price tag of an efficient, low-cost program like CDCom, is unlikely to be successful in the near future in areas of dense poverty. In these areas, any increased cost is a significant barrier to the care of symptomatically silent chronic disease. Therefore, until economic development reaches rural areas, national governments and their third-party collaborators will either need to increase funding for NCDs significantly, pay much more downstream for care of cardiovascular complications, or tether families and local economies to the predictable loss of income and productivity that such care will require.

## Supporting information

S1 Appendix Enrollment form for hypertension. (DOCX)

S2 Appendix Scripts used at CDCom by VHWs: Hypertension and diabetes. (DOCX)

S3 Appendix Example patient chart. (DOCX)

S4 Appendix Risk-based medication prioritization. (DOCX)
