# Supplementary figures and images for: Chronic Disease in the Community (CDCom) Program: Hypertension and non-communicable disease care by village health workers in rural Uganda
Source: PLoS One. 2021 Feb 25;16(2):e0247464. doi: 10.1371/journal.pone.0247464 (PMC7906377; doi:10.1371/journal.pone.0247464)

**S1 Appendix:** Enrollment form for hypertension


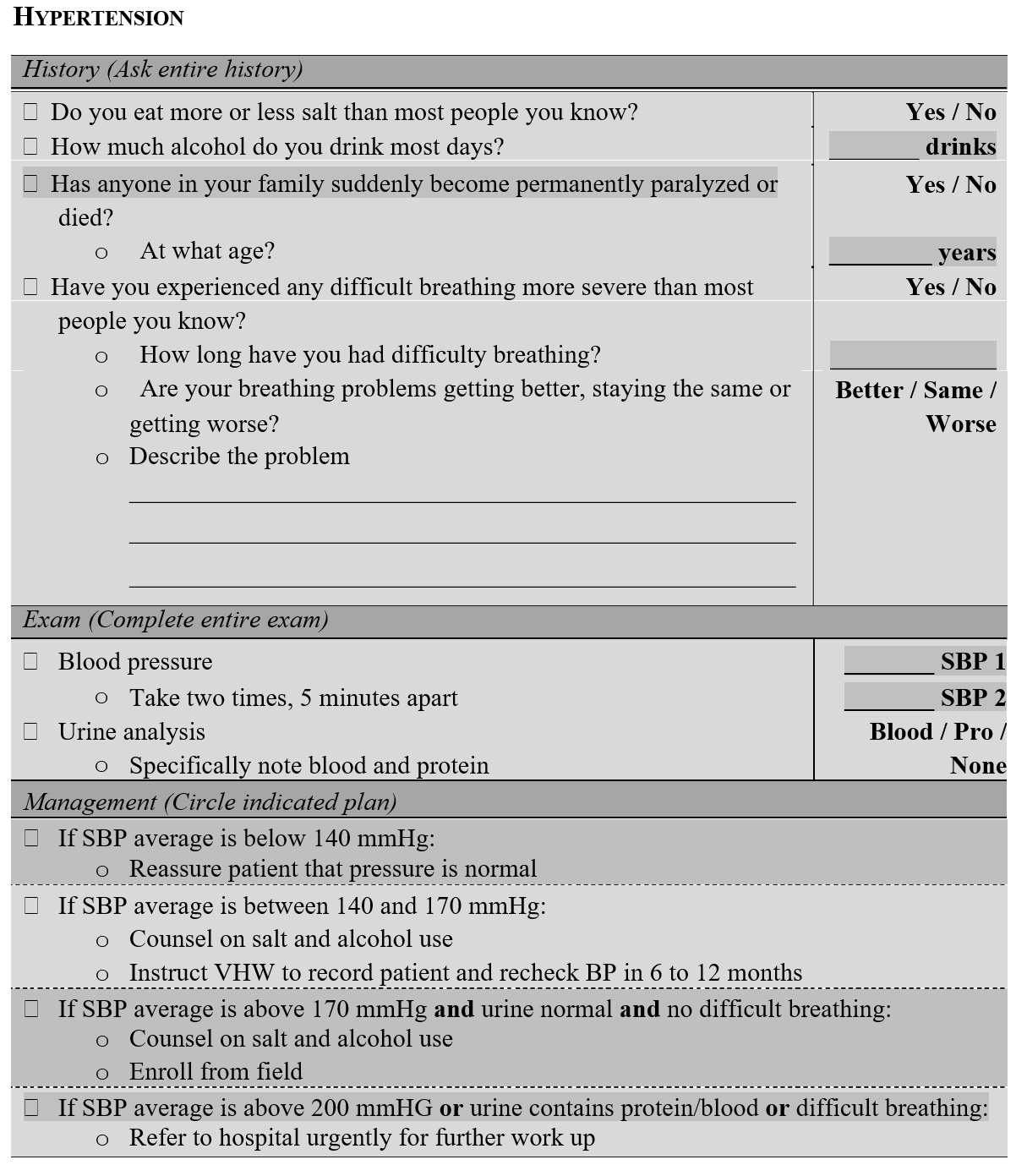

Supplement: S1 Appendix — (DOCX) [file pone.0247464.s001.docx]

**S3 Appendix:** Example Patient Chart


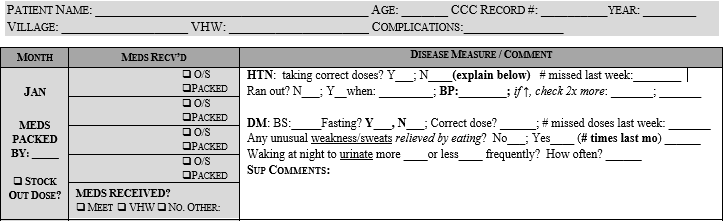

Supplement: S3 Appendix — (DOCX) [file pone.0247464.s003.docx]

**S4 Appendix:** Risk-based medication prioritization


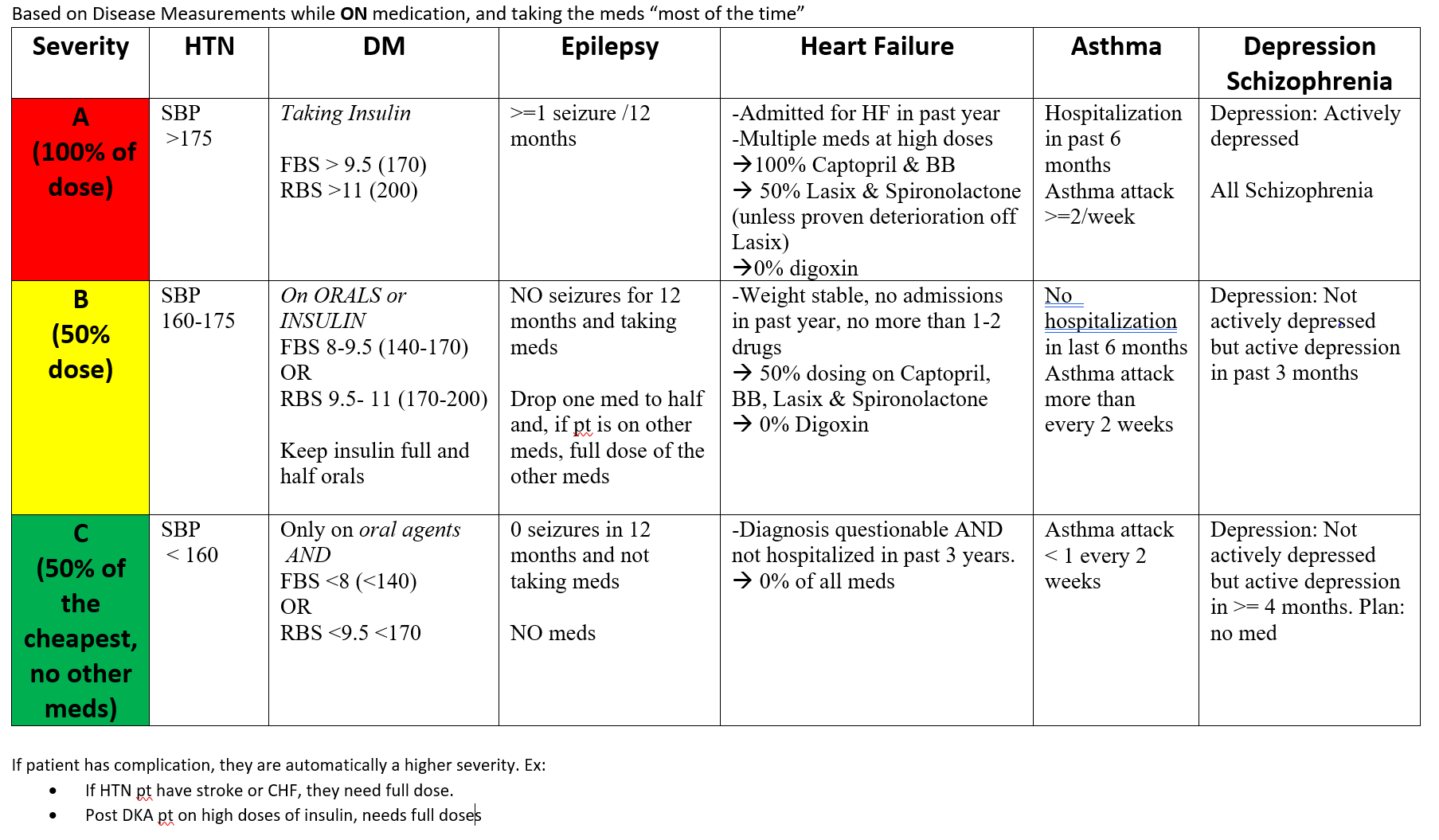

Supplement: S4 Appendix — (DOCX) [file pone.0247464.s004.docx]
