# Supplementary material for: Chronic Disease in the Community (CDCom) Program: Hypertension and non-communicable disease care by village health workers in rural Uganda
Source: PLoS One. 2021 Feb 25;16(2):e0247464. doi: 10.1371/journal.pone.0247464 (PMC7906377; doi:10.1371/journal.pone.0247464)
Supplement: S2 Appendix — (DOCX) [file pone.0247464.s002.docx]

**S2 Appendix:** Scripts used at CDCom by VHWs: Hypertension and Diabetes
